# Enhancing Therapeutic Collaboration in the Care of Substance-Dependent Mothers and Their Children

**DOI:** 10.1192/j.eurpsy.2025.1642

**Published:** 2025-08-26

**Authors:** I. Farmakopoulou, S. Kosmopoulos, M. Theodoratou

**Affiliations:** 1 Sciences of Education and Social Work, University of Patras, Patras; 2 Medical School, National and Kapodistrian University of Athens, AthensPatras; 3 Social Sciences, Hellenic Open University, Patras, Greece

## Abstract

**Introduction:**

Treating substance-dependent mothers and their children presents unique challenges in psychiatric and psychotherapeutic practice. These cases require intensive, coordinated interventions that span mental health, social support, and medical care. Substance dependence in mothers can disrupt early attachment, increase psychiatric risk in children, and challenge the therapeutic alliance. Effective intervention demands seamless interdisciplinary collaboration to provide holistic care that supports recovery and strengthens maternal-infant bonds.

**Objectives:**

This study examines the role of interdisciplinary collaboration in psychiatric and psychotherapeutic care for substance-dependent mothers and their children. Specifically, it aims to assess how collaborative frameworks between psychiatrists, psychotherapists, and allied mental health professionals impact therapeutic outcomes, focusing on reducing burnout and enhancing resilience among care providers.

**Methods:**

A cross-sectional survey was conducted with 91 mental health professionals across 12 agencies involved in the care of substance-dependent mothers and their children. Participants completed an electronic questionnaire assessing demographic data, collaboration experiences, and perceptions of treatment effectiveness. The Maslach Burnout Inventory (MBI-22) and Resilience Evaluation Scale (RES) were utilized to measure burnout and resilience, exploring their influence on therapeutic collaboration and patient outcomes.

**Results:**

Findings show that interdisciplinary collaboration positively correlates with enhanced resilience and lower burnout among professionals, factors crucial in maintaining therapeutic efficacy. Significant associations were found between demographic factors—such as age, gender, and years of service—and both resilience and burnout. Increased resilience appeared to support deeper therapeutic engagement, while low burnout levels were associated with sustained therapeutic consistency.

**Conclusions:**

Interdisciplinary and interagency collaboration is essential in the psychiatric and psychotherapeutic treatment of substance-dependent mothers and their children. Resilience fosters more effective therapeutic engagement, while low burnout levels support sustainable practice. These findings underscore the value of collaborative, resilient mental health teams in achieving positive psychotherapeutic outcomes for this vulnerable population.

**Disclosure of Interest:**

None Declared

